# Tackling Health Inequities: A Unique, Asynchronous Course Designed Through Peer-to-Peer Methods

**DOI:** 10.1177/23821205231203917

**Published:** 2023-10-03

**Authors:** Alexander Burdzy, Michelle Dai, Veda Nagubandi, My Nguyen, Carly Marten, Jennifer Paul-Quinn

**Affiliations:** 1 Penn Pre-Health Programs, University of Pennsylvania, Philadelphia, Pennsylvania, USA; 2 219177 College of Osteopathic Medicine, Touro University Nevada, Henderson, Nevada, USA; 3 487206 Sage Bionetworks, Seattle, Washington, USA; 4 12264 School of Medicine, University of Maryland, Baltimore, Maryland, USA

**Keywords:** health disparities, systemic inequity, social determinants of health, prehealth education, medical education, cultural competency in healthcare, cultural humility, online curriculum

## Abstract

**OBJECTIVES:**

This study investigates the efficacy and feasibility of an asynchronous, peer-to-peer health disparities enrichment course on postbaccalaureate prehealth students’ knowledge, behaviors, and reaction to course materials.

**INTRODUCTION:**

Growing awareness of social inequities has prompted educators of prehealth and medical students to explore student education by addressing systemic healthcare issues. This cross-sectional study assessed reactions, learning, and self-reported behavior changes in students after taking the course “Social Determinants, Disparities, and Preparing for the Future of Healthcare” (SDDH).

**METHODS:**

The curriculum was designed by prehealth postbaccalaureate students for their peers. Course goals were to educate participants on social determinants of health and to build cultural and structural competence in their roles as future healthcare professionals. SDDH is an asynchronous, noncredit-bearing, 5-h online course with 10 modules covering various topics. The Kirkpatrick Model was used to assess the effectiveness of the curriculum, alongside qualitative and quantitative analyses of student performance.

**RESULTS:**

Out of the 102 active students in the prehealth program that accepted the invitation to join, 29 students successfully completed the course (rate of completion = 28%). On average, students expressed positive reactions and attitudes toward the course and experienced an observable increase in knowledge assessment scores upon curriculum completion (*P*-value = .0002). Students’ self-reported observations demonstrated sustained behavioral change 3 months after course completion.

**CONCLUSION:**

It is critical to educate prehealth students on health disparities, structural, and cultural competence. A course such as SDDH may help prehealth students build effective communication skills for advocacy and develop an empathetic, patient-centered approach earlier on in their career pursuit. Some barriers to students completing the entire course include its length, uncredited status, and voluntary self-enrollment.

## Introduction

Health disparities and systemic inequities pose a major threat to the livelihood of Americans.^1^ Data have shown that people of color fare worse than white individuals in a multitude of measures, including infant mortality, chronic conditions, and overall mental and physical health.^2^ Unfortunately, the gaps in quality and availability of healthcare received by minority groups have remained stagnant or even worsened over the past two decades.^3,4^ For example, recent studies indicate poorer outcomes on out-of-hospital cardiac arrests for racial and ethnic minority individuals, who receive less bystander CPR and face more disparities surrounding postresuscitation care.^5,6^ Left unaddressed, these disparities and systemic injustices will cost the United States upward of $1 trillion in annual spending.^7^

The Centers for Disease Control and Prevention (CDC) seeks to attain health equity by eliminating preventable health disparities.^8^ Physicians play a major role in exacting this by demonstrating cultural and structural competence.^9^ Cultural competence is described as the “ability of systems to provide care to patients with diverse values, beliefs, and behaviors, including tailoring delivery to meet patients’ social, cultural, and linguistic needs.”^10–13^ Implicit and explicit biases by physicians perpetuate racism and other inequities, leading to poorer patient–clinician communication and disparities between minority groups and white populations in liver transplant listing and asthma outcomes.^14–19^ Structural competency is defined as a provider's knowledge of how healthcare systems, food access, infrastructure, zoning laws, and medicalization can impact patient health outcomes.^20,21^ Most physicians do not believe that addressing social issues leading to health disparities is their responsibility, and many are apt to blame patient ailments on individual shortcomings or behaviors as opposed to larger structural forces.^22–24^ One way to address these beliefs, attitudes, and awareness is through the incorporation of cultural and structural competence in the education of future providers.

Medical school curricula typically include cultural competency and social determinants of health (SDOH) during preclinical years, with a growing effort to include aspects of structural competency.^15,25,26^ Early exposure to these competencies and social justice is important in the prehealth curriculum, as is emphasized by the American Association of Medical Colleges.^27,28^ A study of Vanderbilt University's Medicine, Health, and Society program showed that health science courses combined with medical humanities emphasizing the importance of structural competency provided better preparation than conventional premedical studies alone.^29^

Postbaccalaureate premedical programs prepare degree-holding students for graduate medical education, thereby presenting an avenue for implementing educational courses on SDOH, cultural, and structural competency.^30,31^ Long-term studies show that graduates of these programs who complete medical school are more likely to work in areas with underserved populations or at institutions that deliver accessible care to vulnerable populations.^32^ Drawing upon a conviction to address health disparities through education at the prehealth level, and the efficacy of the Kirkpatrick Model in evaluating student learning, our course development team at the Penn Pre-Health Post-Baccalaureate Program (PHPB) of the University of Pennsylvania (Penn) created “Social Determinants, Disparities, and Preparing for the Future of Healthcare” (SDDH), a course introducing these important topics to prehealth students.^29,33^

### Program creation and design

**Figure 1.**
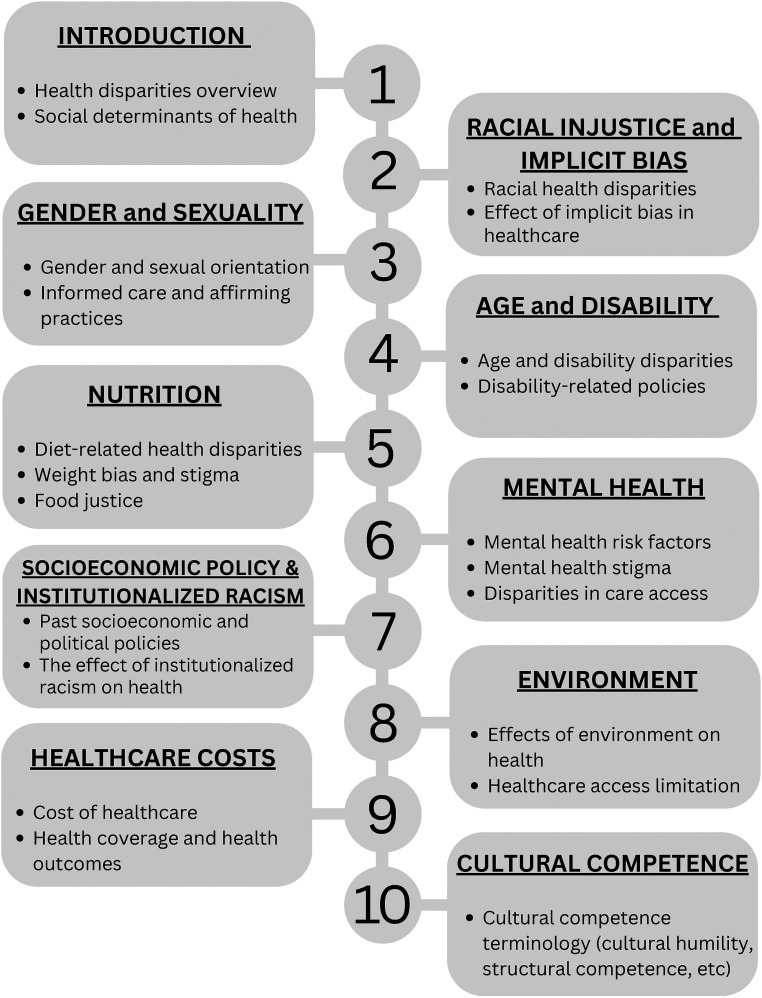
SDDH modules.

Our course development team comprised eight PHPB students. We developed an online, asynchronous course covering topics on SDOH, implicit biases, and healthcare inequities (Figure 1). By laying down a solid foundational understanding of these topics, our goal was to create a cohort of prehealth students who would effect positive change in health disparities as future physicians. In the summer of 2021, our team prepiloted this course through Penn Summer Prep (PSP), a program for high school students to enroll in undergraduate-level classes in Penn's Liberal Arts and Science curriculum. Feedback from students in the PSP program was largely positive, with 93% of students saying the course met their expectations. Utilizing the feedback from PSP, our team then created a self-directed, asynchronous, online, noncredit course titled “SDDH.”

10 modules with brief descriptions of module content. These were completed in sequential order on Canvas to progress through the course. Abbreviation: SDDH, Social Determinants, Disparities, and Preparing for the Future of Healthcare.

A survey was administered to the PHPB student body asking what topics students would like to learn more about. SDDH development began in the fall semester of 2021, and the course debuted in the spring semester of 2022. From the survey results and PHP module feedback, SDDH totaled 10 modules covering the topics detailed in Figure 1. Members of the course development team used their own backgrounds and experience to inform the creation of modules. For example, the “Nutrition” module was created by a registered dietitian with a master's in public health. Certain important topics, such as maternal health, did not have an explicitly dedicated module but were interwoven into other modules. Content for each module referenced peer-reviewed articles or evidence-based sources, such as the CDC and the Kaiser Health Foundation.

Many PHPB students worked part-time in clinical settings and would go on to become health professionals. Therefore, we strove to include application alongside theory by providing employable strategies and action items relevant to a clinical setting. For example, gender-affirming language recommendations were provided in “Gender Inequities,” resources for finding low-priced prescription medications or coupons were referenced in the “Healthcare Costs” module, and “Nutrition” provided recommendations for less stigmatizing practices in the context of weight bias among physicians.

With technical assistance from the Penn College of Liberal and Professional Studies Online Learning Team, SDDH was delivered through the educational online platform Canvas (Canvas Release 2022-01-15). Each module consisted of lecture videos, case studies, recommended readings, and required readings. Prerecorded lecture videos were hosted on Panopto (Panopto v.13.10.1.00001). Modules contained multiple videos, each ranging from 3 to 5 min in length but never spanning more than 15 min in total module playtime. Video lectures featured a PowerPoint presentation (Microsoft PowerPoint v.2211) and a talking head to improve student engagement.^34^ Lecture slides were made available to students on Google Slides (Google LLC v.1.2022.02200), through which students could download the slides in formats best suited to their study needs. The total runtime of SDDH was capped at 5 h to accommodate busy student schedules. Students could start and stop the course at their own leisure and had approximately four months to complete it. Active PHPB students were invited by email to voluntarily self-enroll in the free course.

### Study objectives

This study aims to demonstrate the efficacy and feasibility of a program like SDDH in teaching about health disparities and SDOH. To demonstrate this, we assessed student reactions to SDDH and quantified their learning from the course by measuring changes in knowledge, attitudes, and behaviors.

## Methods

### Course timeline and study population

**Figure 2.**
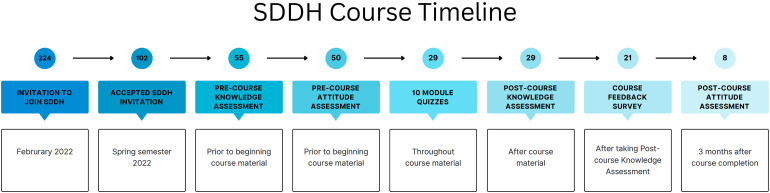
SDDH course timeline.

SDDH debuted in the spring semester of 2022. In February, all active students in PHPB were sent a digital invitation to join SDDH on Canvas. Of the 224 students invited, 102 students accepted the invitation. Fifty-five students began the course (defined as taking the Pre-course Knowledge Assessment), and 29 total students completed all mandatory aspects of the course (defined as completion of Pre- and Post-course Knowledge Assessments and all module quizzes). Twenty-one students filled out the Course Feedback Survey, and eight students responded to the Postcourse Attitude Assessment. The course was closed at the end of the spring semester on 6 June 2022. Details are shown in Figure 2. Throughout the semester, reminders were regularly sent on canvas to encourage participation, and discussion boards were used to facilitate discourse on SDDH topics and answer any student inquiries.

Events are arranged in the order of course progression, from left to right. Colored boxes (middle) indicate a particular event or assessment. Colored circles (top) indicate the number of students who engaged in the corresponding event or assessment. Uncolored boxes (bottom) indicate the absolute or relative timing at which the event took place. Abbreviation: SDDH, Social Determinants, Disparities, and Preparing for the Future of Healthcare.

### Kirkpatrick model and evaluation process

**Figure 3.**
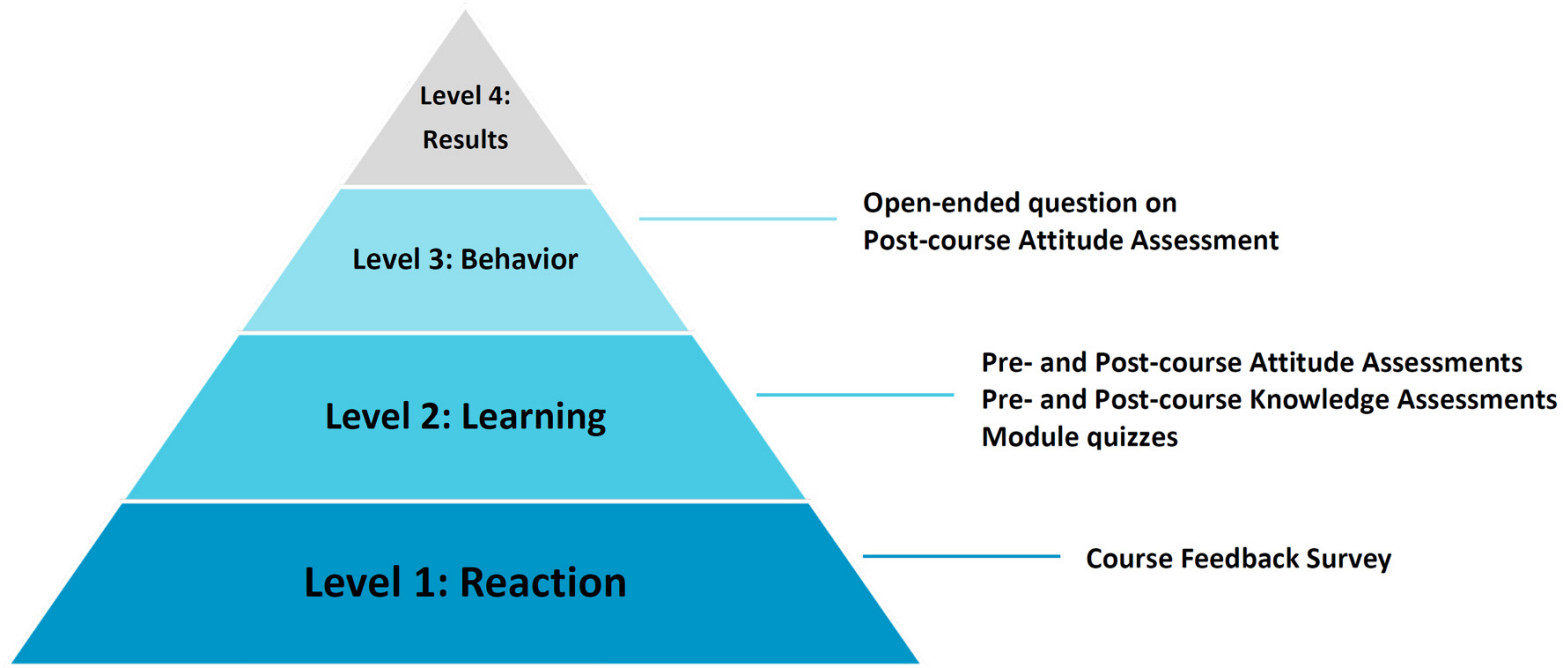
Hierarchical levels of the Kirkpatrick Model of Evaluation with associated assessments.

To track student progress and measure the impact on participant learning and perspectives, we deployed a series of evaluations based on the Kirkpatrick Model, a validated evaluation tool that can assess training program outcomes.^33^ This hierarchical tool comprises four levels, as shown in Figure 3. Our study evaluated the first three levels. Level 1 (reaction) analyzes the extent to which participants find training material relevant and useful. Level 2 (learning) measures the extent to which participants improved knowledge, attitudes, or skills based on course completion. Lastly, Level 3 (behavior) measures the extent to which participants apply their new knowledge and skills to real-life situations.^35^ Level 4, which is the degree to which performance indicators change after the training program, will be assessed at a later time period.

Our evaluation quantified the first three levels of the Kirkpatrick Model. The corresponding SDDH assessments used to measure level-specific outcomes are shown to the right of the respective Kirkpatrick level. Level 4 will be assessed at a later time period. Abbreviation: SDDH, Social Determinants, Disparities, and Preparing for the Future of Healthcare.

To obtain feedback for Level 1, we administered a Course Feedback Survey (Supplemental Material 1.1) immediately following student completion of SDDH, which included questions about how useful participants found course content. The Course Feedback Survey was adopted from a similar questionnaire developed by Rivera et al and was delivered via Canvas.^36^

To measure Level 2, we deployed a Knowledge Assessment (Supplemental Material 1.2) to students prior to beginning the course (precourse), and then again after completing all 10 modules (postcourse). The knowledge assessment was a cumulative 10-question quiz consisting of one question from each module topic (Figure 1). The Pre-course Knowledge Assessment established a performance baseline for which the Post-course Knowledge Assessment could be compared for gauging knowledge acquisition. Additionally, each module had a brief three-question quiz following the lecture content. Students had to receive a passing score of 66.6% (two out of three correct) to progress to the next module. These brief quizzes contained questions about the covered module content and can be referenced in Supplemental Material 1.3. Since these module-specific quizzes were not designed to be formal assessments, but rather serve as quick knowledge checks, students were allowed unlimited attempts, with the highest score recorded. Administration of the Knowledge Assessment occurred through canvas.

Measuring Level 2 also involved the administration of Attitude Assessments. Similarly to the Knowledge Assessments, the Attitude Assessments were delivered once prior to course initiation (precourse), and again 3 months after course completion (postcourse). The Attitude Assessments were based on surveys from Vandana et al^2^ and comprised the same items (Supplemental Materials 1.4 and 1.5). The Pre-course Attitude Assessment set a baseline for participant attitudes that allowed for the quantification of postcourse changes in attitudes and behaviors.

Level 3 of the Kirkpatrick Model was assessed through an additional short-form prompt on the Post-course Attitude Assessment that was not present in the precourse version. This question required participants to describe an instance where they recognized course values playing out in their own behaviors or identifying where they applied course material in their daily lives. The Precourse Attitude Assessment was hosted on Canvas, and the Postcourse Attitude Assessment was sent out via Qualtrics (2022, Provo, UT).

### Data analysis

The assessments and surveys used in this mixed-methods study contained varying amounts of quantitative and qualitative components for each level of the Kirkpatrick Model. For clarification, we have separated the descriptions of these different methodologies below, but our results will be organized by Kirkpatrick level, each involving multimethodological analyses.

### Qualitative analysis

Qualitative survey data was collected as part of the Course Feedback Survey (Supplemental Material 1.1). We analyzed the open text survey data using conventional content analysis,^37^ and themes were generated inductively. This method of analysis was determined to be the best fit for the data, which is primarily intended to inform the improvement of the course rather than test a thematic framework. Correspondingly, the usage of computer-assisted qualitative data analysis software was not necessary for this amount of data, and a digital whiteboard platform, Miro version 0.7.8 (Miro, RealtimeBoard Inc., San Francisco, CA), was used. Respondent identifiers were removed and replaced with randomly generated alphanumeric codes.

### Quantitative analysis

The quantitative analysis involved all levels of the Kirkpatrick Model utilized in the study. In the Course Feedback Survey as well as the Pre- and Postcourse Attitude Assessments, responses were analyzed by the proportion of students choosing a particular response option. Graphical displays of stacked divergent bar charts were generated, which showed the number of students in each of the five Likert response categories. This also allowed for observation of shifting proportions of students in agreement or disagreement prior to starting the course compared to after finishing the course. 49 Precourse and eight Postcourse Attitude Assessments were analyzed in this study.

For the Pre- and Postcourse Knowledge Assessments, student scores were obtained from the Canvas grade book and deidentified with randomly generated alphanumeric codes. This allowed for Postcourse Knowledge Assessments to be compared with their Precourse counterparts for each individual student that completed both (n = 29). The difference between scores was computed as the Precourse Knowledge Assessment score subtracted from the Postcourse Score (post–pre). A one-sample, one-tailed t-test was then performed with a null hypothesized mean value of 0 to verify that the mean difference was positive and statistically significant at α = 0.05. Pre- and Postcourse Knowledge Assessment scores were also depicted in the form of a Sankey diagram, visualizing the proportions of students that experienced a certain score change (SankeyMATIC, 2023).

The 10 module-specific quizzes served as a knowledge check for students, who were allowed to attempt the quizzes as many times as necessary to receive a passing score. Further analysis of these quizzes has been excluded from this study.

Data acquisition, organization, and curation were done using Microsoft Excel (Microsoft Excel v.16.67), and JMP Version 16.0 (JMP, SAS Institute Inc., Cary, NC, USA) was used to perform statistical analysis and generate figures.

## Results

### Kirkpatrick model results

#### Level 1: Reaction

**Figure 4.**
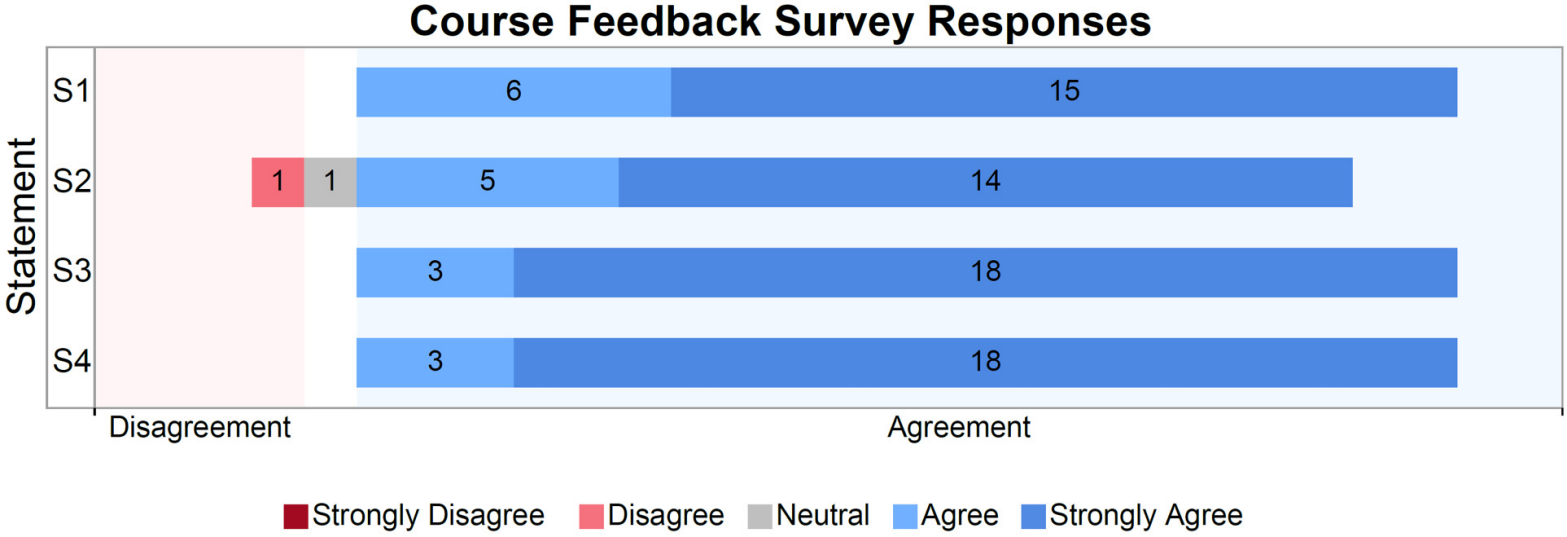
Course feedback survey responses.

The four statements that were used to assess students’ reactions in the Course Feedback Survey can be referenced in Supplemental Materials 1.1. Of the 224 students invited to take the course, 102 students accepted the invitation to take it, and a total of 29 students completed the curriculum. Out of the 29 students who completed the course, a total of 21 feedback surveys were collected (response rate = 72%). The number and proportion of student responses to each statement are shown in Figure 4. Overall, the vast majority of student responses were in agreement, with a greater proportion of students choosing “Strongly Agree” than any other item option for every one of the 4 statements. Data collected in tabular form can be referenced in Supplemental Table S1.

Student responses to each of the four statements in the Course Feedback Survey are depicted as a stacked divergent bar chart (n = 21). Numerical labels on colored bars represent the number of students who picked the corresponding Likert response item depicted in the legend at the bottom of the figure. Refer to Supplemental Material 1.1 for more details on the four statements used.

Qualitative findings corroborate the trend in strong agreement with the items above. Survey participants appraised the course as informative and useful:

*I really liked the hand-picked journals/readings! Definitely helped broaden my knowledge!*

(Participant DYN)

*I loved all of the supplemental papers and case studies that were included. They gave such strong support and examples of everything being taught in this course.* (Participant ZAG)

#### Level 2: Learning

Fifty-six students completed the Precourse Knowledge Assessment and 29 students went on to complete the Postcourse Knowledge Assessment, indicating an attrition rate of 48%. Knowledge assessment results were analyzed only for students who completed both assessments (n = 29). The Knowledge Assessment was graded on a 10-point scale and can be referenced in Supplemental Material 1.2.

**Figure 5.**
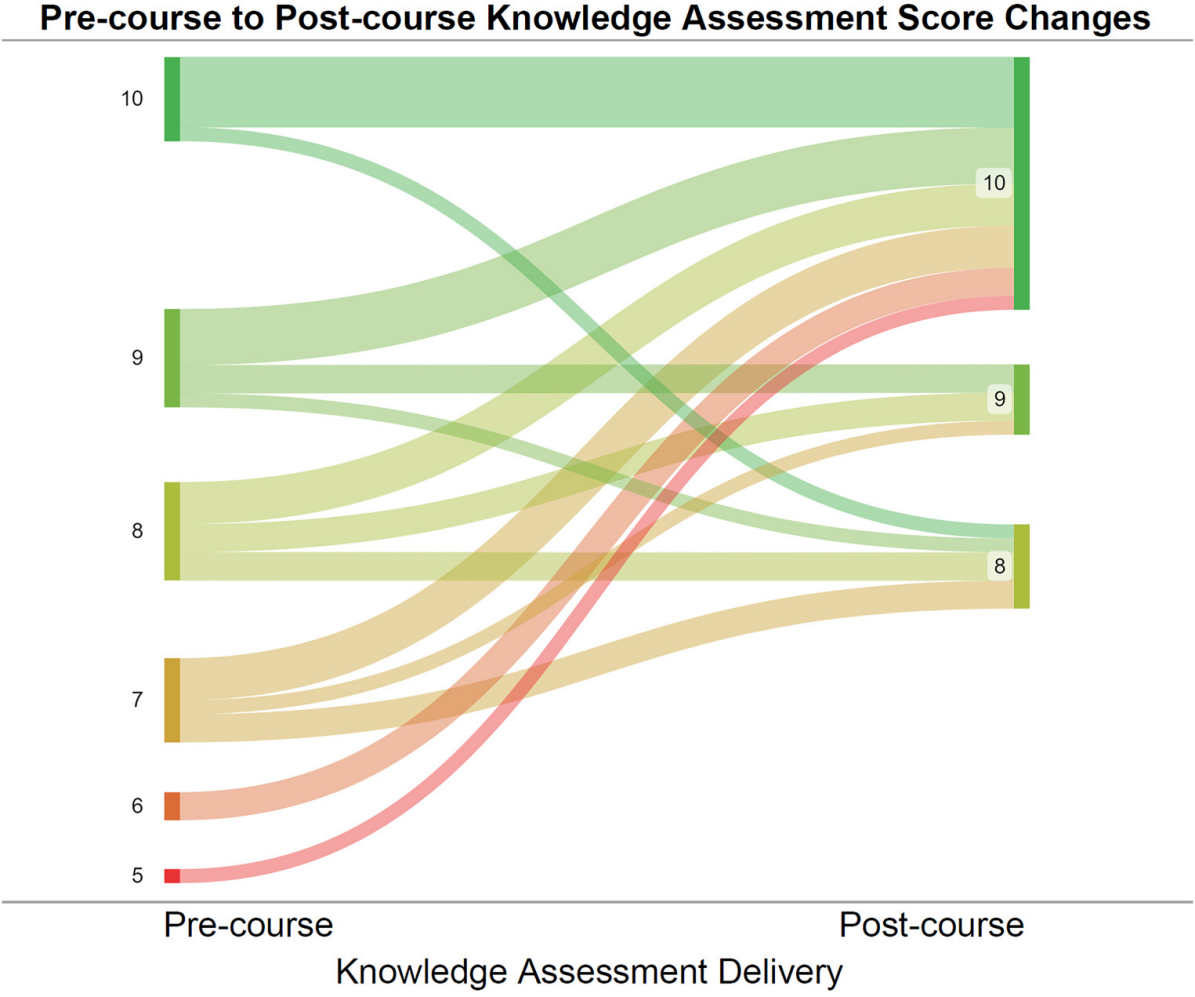
Precourse knowledge assessment scores linked to postcourse results (n = 29).

The precourse mean performance on the Knowledge Assessment was 8.2 points, and the postcourse mean was 9.4 points. On average, students showed a score increase from the pre to postcourse knowledge assessment of 1.2 points. A one-tailed, one-sample t-test performed on the score differences confirmed that the score improvement was indeed significant (*P* = .0002, n = 29). To investigate how this improvement was distributed, score changes were visualized in Figure 5. Notably, 62% of students (n = 18) experienced a score increase, 31% (n = 9) received the same score and only 7% of students (n = 2) experienced a score decrease.

Left: Scores prior to beginning the course material. Right: Scores after completing the course. Connecting lines’ thicknesses denote the number of students who received the respective scores on the knowledge assessments. Score increase: 62% (n = 18). No change: 31% (n = 9). Score decrease: 7% (n = 2). Refer to Supplemental Material 1.2 for the questions used in the Knowledge Assessment.

Additionally, we measured the lasting change in students’ attitudes surrounding health disparities by quantifying their responses on the Postcourse Attitude Assessment 3 months after the program had ended. The four statements used in the analysis can be found in Supplemental Material 1.5.

**Figure 6.**
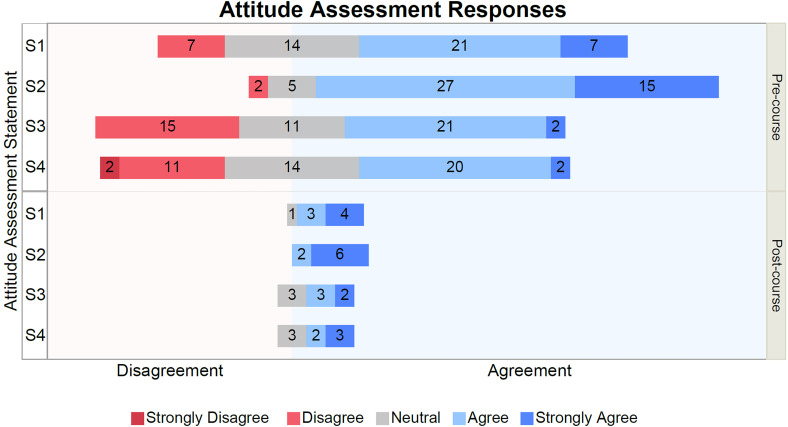
Attitude assessment responses before and after the course.

A comparison of students’ Precourse and Postcourse Attitude Assessment responses is shown in Figure 6. Of the 49 students who completed the Precourse Attitude Assessment, eight students responded to the Postcourse Attitude Assessment, signifying an attrition rate of 84%. Overall, each one of the four statements (Supplemental Materials S1–S4) showed an increased proportion of agreement during the postcourse administration of the survey. Notably, there were no disagreeing responses on any of the statements on the Postcourse Attitude Assessment. The proportion and number of students responding to the Likert scale options for each question is available in tabular format in Supplemental Table S2.

Top: Precourse ratings (n = 49). Bottom: Postcourse ratings (n = 8). Overall, all four statements showed increased proportions of students in agreement and decreased proportions of students in disagreement 3 months after course completion. Refer to Appendices 1.4 and 1.5 for more details on the four Attitude Assessment statements.

#### Level 3: Behavior

Added to the end of the Postcourse Attitude Assessment was an open-text opportunity for students to reflect on how they had operationalized course concepts into their daily lives. Their responses indicate the ways in which students are applying their learning, particularly in the workplace. Respondents reflected on the relationship between the COVID-19 pandemic and health equity:

*When I worked in a […] COVID clinic […], I saw firsthand how COVID disproportionately affects minority groups. I heard patients mention how it was hard for them to find transportation and time away from work to go to a clinic, which directly affects their access to healthcare.* (Participant X5U)

While it cannot be determined when this participant first applied a health equity lens to the COVID pandemic, the content in the SDDH course encouraged students to take a structural approach in understanding disproportionate disease burdens.

Participants’ structural analysis extended to assessing interventions in their hospital workspaces aiming to meet the needs of marginalized patients:

*[A teaching hospital in a large East Coast city] offers in-person translators, free food, and any additional assistance to ensure all patients get the care they need. I appreciate this course for providing me with more background on health disparities and it has allowed me to identify more of these issues.* (Participant BWK)

In addition to attesting, in their own words, to the effect of the course, this participant demonstrates a public health approach that we sought to cultivate in students of the course, connecting language access and nutrition assistance with patient health.

### Independent qualitative results

As mentioned previously, qualitative results were also gathered from the Course Feedback Survey's open-text questions (Supplemental Material 1.1). A full list of themes and exemplar data can be found in Supplemental Table S3. In response to the question *What action item(s) do you plan to take in the future as a result of this course?*, respondents’ answers largely related to the awareness and introspection of health disparities that they plan to maintain as a result of this course. Participants envisioned educating others on what they learned and imagined how these lessons would impact their future clinical practice. Of particular note by respondents was a desire to build on education regarding gender-affirming care:

*Learning more about gender-affirming healthcare since I honestly haven’t heard of this term until I took this course.* (Participant 0CR)

In response to the question *What do you see as potential barriers to applying what you have learned in the real world?*, participants did anticipate interpersonal barriers, such as lack of support from colleagues, but the barriers identified were primarily structural in nature, such as legal and legislative barriers as well as the negative influence of financial and profit-driven motives in healthcare:

*Healthcare system in general is more of a business rather than wanting to actually provide help to patients so the unequal access to healthcare with [sic] prevent me from applying what I learned.* (Participant N0B)

Respondents also envisioned organizational barriers, especially those which interact with their social or economic position:

*I think my own identity may be a barrier to applying this in the real world as I try and navigate my own experience of health inequity.* (Participant QCG)

*The balance between paying off student loans and working for lower paying jobs that would allow me to better serve my commute [sic] members.* (Participant US3)

In response to the final open-text question, *What did you like best about the course and how can we improve the course?*, respondents were highly complimentary, noting their appreciation for the course's self-paced style and inclusion of case studies. Participants’ recommendations for improvement involved adding an interactive component such as discussion sessions or inviting live speakers.

## Discussion

Health disparities are a growing threat to the American healthcare system. The results from our analysis demonstrate how a program that discusses, examines the causes of and provides solutions to these issues can be a promising way to enlighten prehealth postbaccalaureate students. Additionally, it is an avenue for students to start making changes in their daily lives based on the knowledge they have gained. Students showed a statistically significant gain in knowledge upon course completion. From the feedback gathered, students were able to demonstrate a positive change in attitudes and continued behavior after taking the SDDH course.

Students’ overall positive reaction to the course suggests that there is an appreciation for understanding the SDOH, and health inequities, and incorporating their newfound insight into their daily lives. We found that SDDH positively impacted attitudes toward the learning of healthcare disparities and contributed to participants’ increased awareness of SDOH. In respondents’ self-assessments of whether they witnessed themselves applying course concepts, students reported adopting an empathetic and patient-centered approach. They also identified extrinsic and intrinsic barriers that may inhibit their application of course concepts. From our qualitative data, we see that students experienced heightened awareness and introspection inspired by the course.

Over half (53%) of students who started the course (n = 55) continued to completion (n = 29). Among the students that accepted the course invitation (n = 102), the completion rate was 28%, higher than other similar studies reporting rates of participation in voluntary, online courses.^38,39^ This completion rate reflects the optional, uncredited nature of the course, with limited incentives for students other than their own interests. Eighty-three percent of the PHPB student population work at least part-time, with most maintaining full-time employment outside their coursework.^40^ These circumstances present additional constraints on students’ ability to complete SDDH alongside a rigorous STEM course load. While SDDH occupies a unique space in terms of its structure, voluntary online classes such as massive open online courses have a median completion rate of 12.6% in comparison.^39^ However, the low rate of conversion from course acceptance to course completion is cause for further investigation.

The costs of the project, time commitment, and resources needed were modest. Students who developed the course were paid a stipend of $500, totaling $4000, for their efforts. Neither the outside subject matter expert who reviewed the course nor the Penn Online Learning Team were paid for their support of the project. It took the students 6 months with approximately 3 h a week devoted to the project to complete it from inception to launch. As digital learning continues to grow in its ubiquity, designing and creating a self-paced, online course has become streamlined with platforms such as Canvas and Panopto. Individual programs seeking to take on similar endeavors will find that most of the time and cost involved with creation will be dependent on the breadth and depth of materials being covered in the course. Our efforts have shown that a short introductory course could be fully prepared in less than half a year's time by a team of motivated individuals. We hope that these results can inspire other programs to build solid educational foundations in social justice, broadening the scope of prehealth student preparation to keep up with expanding graduate program expectations.

### Limitations

While student feedback about SDDH was promising, our research evaluation does have limitations. First, enrollment in the course was optional. Students who were already interested in or had background knowledge about health disparities may have been more likely to self-enroll in the course. Consequently, this may have introduced selection bias and inflated the results of knowledge and attitude assessments. Caution should also be taken with the interpretation of behavioral self-reporting. Furthermore, 55 students initiated the course, 29 students completed it, and among them, only eight students responded to the optional postcourse attitude assessment survey. Our results are therefore limited by small sample sizes, as well as generalizability beyond the prehealth postbaccalaureate student population. The low completion rate also introduces possible nonresponse bias into our results. It is likely that students completing the course and surveys in its entirety may have had a higher affinity for or preexisting knowledge of the topics discussed in the course, leading to a positive skew in both knowledge and attitude assessment results.

Future studies involving larger, more diverse populations of students are warranted. Furthermore, the absence of participant demographic data collection is a limitation. Given the peer-to-peer nature of the educational program, course developers surmised that having anonymized responses would make participants more comfortable with sharing their opinions and feedback. However, this limitation will be addressed in larger future cohorts where the risk of participant reidentification is smaller. Certain topics had to be omitted from the course due to its length of 5 h. Our study was quasi-experimental by conventional design for a pilot program, and thus a control group of students was not available. Evaluating the effectiveness of a peer-to-peer model of teaching over a more traditional framework can be done in future studies with a control group, consisting of students who complete the SDDH assessments and surveys without taking the course. To address selection, attrition, and nonresponse biases, making course enrollment and assessments mandatory may provide more robust results.

The timing of SDDH assessment delivery in the spring semester may also have played a significant role in nonresponse bias and attrition rates. By nature of the population studied, prehealth students become increasingly busy in the spring with taking national examinations for graduate school admissions, such as the Medical College Admission Test or Graduate Record Examination. Additionally, many students devote a significant amount of time in late spring to completing graduate health professions applications. Future results from fall semester cohorts can serve as a comparison to shed light on how the spring semester timing of SDDH may have affected attrition rates.

### Ongoing course development

When building a course covering contemporary topics, it is of utmost importance to maintain the relevance of the curriculum to the present day, while still providing historical examples of issues from the past. To this end, our program is organizing an advisory board of experts and pooling university resources to keep our content up-to-date and well-informed from diverse subject matter experts. With each semester, we seek to expand the diversity of topics addressed. Future course iterations plan to include topics on American Indian/Alaska Native (AI/AN) health, reproductive justice, and immigrant health. Additionally, in response to student feedback from Spring 2022, we implemented optional, synchronous monthly discussions between course facilitators and students over course topics in the Fall of 2022, which led to increased student engagement and thought-provoking discussions. We are testing novel strategies to increase the conversion rate from course acceptance to course completion, including more frequent email messaging and language reflecting the professional development benefits that students can gain from taking the course. New modules will continue to be added, and the course will be designed so that students can choose any 10 modules among the offerings available for course completion. The once-optional Postcourse Attitude Assessment is now a requirement for course completion to mitigate nonresponse bias. Lastly, we are working to add experiential and clinical components by opening community volunteer opportunities and working to partner with local Federally Qualified Health Centers.

## Conclusion

Prehealth education has traditionally centered around building a strong foundation in sciences. While this is necessary, it remains insufficient for nurturing a well-rounded prehealth education. Fostering awareness of cultural backgrounds, open-mindedness to structural change, and humility in understanding healthcare barriers requires complementary education on SDOH, cultural, and structural competence. This course encouraged students to apply their analytical skills beyond the biological pathways of the classroom and into today's ever-changing healthcare ecosystem, thereby brainstorming creative solutions for the future. It is through exacting changes in prehealth education that we seek to create the next generation of healthcare professionals better prepared to close the gaps in American healthcare. We hope that our work serves as both inspiration and a foundational model for other programs to build upon for their students.

## Supplemental Material

sj-docx-1-mde-10.1177_23821205231203917 - Supplemental material for Tackling Health Inequities: A Unique, Asynchronous Course Designed Through Peer-to-Peer Methods Click here for additional data file. Supplemental material, sj-docx-1-mde-10.1177_23821205231203917 for Tackling Health Inequities: A Unique, Asynchronous Course Designed Through Peer-to-Peer Methods by Alexander Burdzy, Michelle Dai, Veda Nagubandi, My Nguyen, Carly Marten and Jennifer Paul-Quinn in Journal of Medical Education and Curricular Development

sj-pdf-2-mde-10.1177_23821205231203917 - Supplemental material for Tackling Health Inequities: A Unique, Asynchronous Course Designed Through Peer-to-Peer Methods Click here for additional data file. Supplemental material, sj-pdf-2-mde-10.1177_23821205231203917 for Tackling Health Inequities: A Unique, Asynchronous Course Designed Through Peer-to-Peer Methods by Alexander Burdzy, Michelle Dai, Veda Nagubandi, My Nguyen, Carly Marten and Jennifer Paul-Quinn in Journal of Medical Education and Curricular Development
