# Supplementary material for: Tackling Health Inequities: A Unique, Asynchronous Course Designed Through Peer-to-Peer Methods
Source: J Med Educ Curric Dev. 2023 Oct 3;10:23821205231203917. doi: 10.1177/23821205231203917 (PMC10563473; doi:10.1177/23821205231203917)
Supplement: sj-docx-1-mde-10.1177_23821205231203917 - Supplemental material for Tackling Health Inequities: A Unique, Asynchronous Course Designed Through Peer-to-Peer Methods [file sj-docx-1-mde-10.1177_23821205231203917.docx]

# Supplementary Material

1. **Evaluation Materials**

## Course Feedback Survey

- - 1. Please rank how much you agree or disagree with the statements below:

|  | Strongly disagree | Disagree | Neutral | Agree | Strongly Agree |
| --- | --- | --- | --- | --- | --- |
| Course met objectives | ⃝ | ⃝ | ⃝ | ⃝ | ⃝ |
| Course was a valuable use of my time | ⃝ | ⃝ | ⃝ | ⃝ | ⃝ |
| Course materials include useful resources | ⃝ | ⃝ | ⃝ | ⃝ | ⃝ |
| I believe that I will apply course info to address health disparities | ⃝ | ⃝ | ⃝ | ⃝ | ⃝ |

- - 1. What action item(s) do you plan to take in the future as a result of this course?
    2. What do you see as potential barriers to applying what you have learned in the real world?
    3. What did you like best about the course and how can we improve the course?

## Pre and Post-course Knowledge Assessment (Answers in bold)

- - 1. Which of the following below best fits with the term ‘health equity’?
       1. Having unequal access to healthcare resources
       2. Giving everyone the same level of assistance to meet their health needs

### Giving individuals varying levels of assistance depending on their health needs

- - - 1. Eliminating health barriers for individuals
    1. In which health conditions do racial disparities exist?
       1. Mental health condition symptoms, severity and persistence
       2. Both maternal and infant health
       3. Infectious diseases, such as HIV

### All of the above

- - 1. What is gender health inequity?
       1. Unequal access to healthcare based on one’s gender, sex, gender identity, and/or sexual orientation
       2. Unequal health outcomes based on one’s gender, sex, gender identity, and/or sexual orientation
       3. Unequal opportunities for health interventions based on one’s gender, sex, gender identity, and/or sexual orientation

### All of the above

- - 1. Which of these describes tenants of the social model of disability?
       1. I. A disability is a medical phenomenon that is fixed or lessened with treatment.
       2. II. People have impairments, but it is the social world that makes someone disabled.
       3. III. The experience of disability is structured by the relationship between disabled people and their environment.
       4. I and III
       5. I only

### II and III

- - - 1. I, II, and III
    1. What effect can childhood weight-related teasing have on the development of eating disorders?
       1. Weight-related teasing in adolescence results in less disordered eating in adulthood.
       2. Weight-related teasing in adolescence has no effect on disordered eating in adulthood.

### Weight-related teasing in adolescence can contribute to the development of eating disorders in adulthood.

- - 1. Identify one risk factor of mental illness.
       1. Peer pressure

### Genetics

- - - 1. Institutional bias
      2. All the above
    1. Which of these following is NOT an example of institutionalized racism?
       1. Studies have shown that patients with darker skin tone of all ages receive lower doses of pain medication compared with white patients in the United States
       2. An employer rejected a Black candidate for a job after meeting her. He was visibly shocked and turned her down flat, without asking about her credentials. When asked what was wrong, he said something about maintaining the company image.

### A Black man recently moved to a wealthy neighborhood with his family. He often encounters people approaching him, asking him if he is looking for somebody in the neighborhood or telling him that he is in the wrong neighborhood.

- - 1. Which one of the following statements is NOT true about the US healthcare system?
       1. Around 17

### The US healthcare system provides universal coverage to all citizens.

- - - 1. There are still relatively few government regulations on how much companies can charge for insurance, drugs, or care.
      2. High-cost, intensive medical intervention is incentivized over primary and preventative care.
    1. Which of the following options encapsulates people of color’s relationship to asthma?
       1. People of color are less vulnerable to asthma due to the environments that they are more likely to live and work in.

### Due to unequal exposure to outdoor and indoor air pollution, people of color are particularly vulnerable to asthma.

- - - 1. All races are equally susceptible to developing asthma due to equal ambient outdoor and particulate matter pollution levels nationwide.
    1. Define cultural competence in and why it is important in healthcare.
       1. Cultural competence is diversity and it is important to care for diverse populations.
       2. Cultural competence is a deep understanding that more than one culture exists and is important to interact with and serve people from different cultures.
       3. **Cultural competence is the recognition that multiple cultures exist that are different from one’s own coupled with the adoption of personal and institutional policies and practices that empower persons to engage with, and serve respectfully and honestly, persons of various traditions and backgrounds.**

## Individual Module Quizzes for Modules 1-10 (Answers in bold)

### Module 1: Introduction to Health Disparities

- - 1. Which of the following is NOT an example of a social determinant of health?

### Having sickle cell anemia

- - - 1. Living next to a factory that emits heavy metals in pollution
      2. Having ten different grocery stores within a 1 mile radius of your home
      3. Making a livable wage
    1. A local agency decides to give every resident in a zip code a $100 voucher to be used at a local health clinic. This kind of assistance is an example of:
       1. Healthcare inequality
       2. Healthcare equality

### Healthcare equity

- - - 1. Healthcare justice
    1. Which of the following diseases below show evidence of health disparities and systemic issues with race:
       1. Heart disease
       2. Diabetes
       3. Asthma

### All of the above

**Module 2: Racial Injustice and Implicit Bias**

1. What is the most prominent difference between institutionalized racism and interpersonal racism?
   1. Institutionalized racism is the discrimination of official laws based on race, while interpersonal racism is the discrimination based on race between individuals

### Institutionalized racism is the discrimination based on race that is embedded in laws, regulations, organization, while interpersonal racism is the discrimination based on race between individuals

- 1. Institutionalized racism is the blocking of access of individuals to certain rights based on racism, while interpersonal racism is the discrimination based on race between individuals

1. Which of the following below is an example of unconscious bias?
2. Crossing the street at night when you see a Black man walking in your direction, without even realizing why you are crossing the street.
3. A Latino student is complimented by a teacher for speaking perfect English, but he is actually a native English speaker
4. Having the notion that girls are more likely to be better in language over math, whereas males are more likely to be better with math than language
   1. I
   2. II
   3. I, and III

### I, II, III

1. Which of the following is an example of a racial health disparity?
   1. A racial group having higher prevalence of a disease
   2. A racial group having a similar prevalence of disease, but a higher rate of mortality
   3. A racial group having a higher severity or more complications association with a particular condition

### All of the above

**Module 3: Gender Inequalities**

1. Label each of the following as either sex, gender, gender identity, or sexual orientation (fill in the blank)
   1. A social construct consisting of a range of characteristics both personal and social that may or may not be related to one’s sex assigned at birth **gender**
   2. A person’s identity with regard to the gender or genders they are sexually attracted to **sexual orientation**
   3. A person’s self-conception of gender, including those social and personal characteristics they identify with and use to express themselves **gender identity**
   4. An assignment at birth based on one’s primary, anatomical, characteristics **sex**
2. Which of the following are health disparities endured by individuals from gender and sex-diverse communities?
   1. Unequal access to healthcare services and resources in comparison to the general population
   2. Unequal access to adequate services that target the needs of persons who are from gender and/or sex-diverse communities
   3. Unequal access to health insurance, or adequate health insurance, that covers the needs of persons from gender and/or sex-diverse communities

### All of the above

1. What is affirming care?
   1. Affirming care is cultural competence. It includes treating persons from sex and gender-diverse communities with dignity and respect.

### Affirming care is a holistic approach to treating persons with dignity and respect regardless of their sex, gender, gender identity, sexual orientation, or other socioeconomic identities.

- 1. Affirming care is good care. It includes being nice, respectful, honest, and ethical.

1. **Bonus question: identify a structural practice that the institution of medicine can adopt to ensure the progress towards health equity for queer community members. Hint: diversity and inclusion. (fill in the blank) **Representation**

### Module 4: Age & Disability

1. Before the COVID-19 pandemic, the life expectancy for white Americans was about 4 years longer than Black Americans. What effect did the COVID-19 pandemic have on this life expectancy gap?
   1. The gap narrowed.

### The gap widened.

- 1. The gap remained unchanged.

1. In the 1921 Supreme Court case Buck v. Bell, the US Supreme Court

### upheld compulsory sterilization.

- 1. struck down compulsory sterilization.
  2. did not make a decision on compulsory sterilization.

1. Which of the following is an example of identity-first language?
   1. I. person with a disability
   2. II. disabled person
   3. III. autistic person
   4. I only

### II and III

- 1. I and III
  2. I, II, and III

### Module 5: Nutrition

1. What is the primary difference between the two types of food insecurity?
   1. Very low food security has a reduced variety of food, while low food security does not.

### Very low food security has a reduced amount of food, while low food security has little or none.

- 1. Very low food security is the same as low food security except for a higher level of each variable.
  2. Very low food security has reduced quality, variety or desirability of diet.

1. Which of the following populations tend to have higher rates of obesity and type 2 diabetes than their counterparts?
   1. Native Hawaiian and Pacific Islander compared to non-Hispanic white individuals
   2. Individuals identifying as LGBTQ compared to individuals who do not identify as LGBTQ
   3. Individuals with disabilities compared to those without

### All of the above

1. Which of the following are some of the negative ways in which weight bias toward patients can affect the practice of physicians?
   1. Physicians may hold stereotypes about higher weight patients, like laziness or noncompliance
   2. Physicians may also over-attribute symptoms to obesity and provide few treatment recommendations outside of losing weight.
   3. Physicians may oversimplify the complex process of weight gain and loss to “eat less, exercise more” or “calories in, calories out.”

### All of the above

**Module 6: Mental Health**

1. Which of the following is a mental health disorder discussed in this module?
   1. Anxiety
   2. Bipolar Disorder
   3. Substance abuse

### All the above

1. Which of the following is an example of toxic stress?
   1. A beloved captain of the soccer team has failed their last 2 Calculus midterms and is going through their parents’ divorce
   2. An aspiring actress misses out on the lead role in the school musical, is worried about the lack of extracurricular activities for her college resume, and turns to her group of friends for guidance

### A new high school student moved from a different state and has struggled to make connections and has been routinely bullied in the cafeteria at lunch

- 1. None of the above

1. What percentage of adults have at least one ACE?
   1. 16%
   2. 42%

### 61%

- 1. 88%

### Module 7: Socioeconomic Policies and Institutionalized Racism

1. Which of the following policies is NOT examples of institutionalized racism?
   1. Internment of Japanese Americans during World War 2

### Affirmative Action

- 1. Standardized testing
  2. School-to-prison pipeline
  3. Redlining

1. Which is TRUE regarding the definition of structural inequality?
   1. Structural inequality refers to how the experiences of people within a particular identity category are qualitatively different from each other depending on their other identities
   2. Structural inequality when social changes disable a whole group of people to move up the social class ladder

### Structural inequality is the condition where one category of people are attributed an unequal status in relation to other categories of people

1. What is the correct order of the different levels of racism from micro to macro level?
   1. Personal → Institutionalized → Interpersonal → Structural
   2. Personal → Interpersonal → Institutionalized → Global
   3. Interpersonal → Personal → Institutionalized → Structural

### Personal → Interpersonal → Institutionalized → Structural Module 8: Environmental Health

1. Why did the Warren county protests propel the environmental justice movement in the US?

### It revealed how areas with racial and economic minorities were often targeted to bear the consequences of environmental pollution

- 1. It showed how the EPA and federal government can collaboratively work with minority communities to come up with solutions to issues of environmental pollution
  2. It demonstrated how all citizens in America are subject to equal amounts of environmental pollution, encouraging everyone to think about their futures

1. True or False: HOLC’s historical discriminatory redlining that fuels stark segregation in Philadelphia today contributes to increased vulnerability of populations of color to COVID-19 through increased case rates, decreased access to testing, and increased structural susceptibility.

### True

- 1. False

1. What are some ways in which environmental health impacts other social determinants of health? Select all that apply.
   1. **Differences in access to sanitation and clean water between low income communities and wealthier communities**
   2. **The difference in air pollution between industrialized cities and rural areas**
   3. **African Americans, Hispanic and Indigenous people face the highest burden of asthma in the United States**

**Module 9: Healthcare system and cost of disparities**

1. Which of the following is the most common reason why nonelderly adults were uninsured? (According to the KFF 2019 National Health Interview Survey)
   1. Do not need or want
   2. Ineligible for coverage
   3. Cannot find a plan that meets needs

### Coverage not affordable

- 1. Lost job

1. Which of the following is a policy-level solution for controlling drug costs?
   1. Discussion with patients about drug affordability
   2. Awareness of resources and information on drug pricing

### Implementing emergency access laws

- 1. Using low-cost generics or biosimilars

1. Which of the following reasons does NOT explain why insulin costs so much?
   1. There are only a few drug companies monopolizing the market, effectively eliminating competition
   2. Patent evergreening prolongs a companies’ monopolizing of a drug formulation
   3. Pharmacy benefit managers and other middlemen markup drug prices

### Insulin is not considered a basic necessity and can therefore be priced however the manufac- turer sees fit.

**Module 10: Cultural competency in healthcare and bioethics**

1. Developing cultural competence on an individual level involves all of the following except:
   1. Demonstrating empathy and willingness to interact with cultural differences

### Adhering to a standard of care in which each patient is treated in exactly the same way

- 1. Having consciousness of cultural differences and accumulating information about other cultures
  2. Consistent reflection on one’s thoughts and actions

1. Which of the following examples would serve as the most effective framework for developing cultural competence?
   1. Individual only, involving the healthcare providers themselves
   2. Organizational level, so that everything underneath an organization will change

### Development on the individual, team, and organizational levels

- 1. Both individuals and organizations must make changes

1. Which of the following Bioethical pillars is cultural competence closely related to?
   1. Justice
   2. Beneficence
   3. Nonmaleficence
   4. Autonomy
   5. **All of the above**

## Pre-course Attitude Questionnaire

- - 1. Please evaluate the following statements based on how much you agree/disagree with them:

Strongly disagree

Disagree Neutral Agree Strongly

Agree

I am confident in my abilities to apply mitiga- tion strategies to health disparities in real-life scenarios.

I am comfortable with saying that I am aware of potential sources of disparities within health systems.

I have experience with identifying solutions that reduce the incidence of health dispari- ties.

My prior training has adequately prepared me to minimize and discuss health disparity issues.

## Post-course Attitude Questionnaire

⃝ ⃝ ⃝ ⃝ ⃝

⃝ ⃝ ⃝ ⃝ ⃝

⃝ ⃝ ⃝ ⃝ ⃝

⃝ ⃝ ⃝ ⃝ ⃝

- - 1. Please evaluate the following statements based on how much you agree/disagree with them:

Strongly disagree

Disagree Neutral Agree Strongly

Agree

I am confident in my abilities to apply mitiga- tion strategies to health disparities in real-life scenarios.

I am comfortable with saying that I am aware of potential sources of disparities within health systems.

I have experience with identifying solutions that reduce the incidence of health dispari- ties.

My prior training has adequately prepared me to minimize and discuss health disparity issues.

⃝ ⃝ ⃝ ⃝ ⃝

⃝ ⃝ ⃝ ⃝ ⃝

⃝ ⃝ ⃝ ⃝ ⃝

⃝ ⃝ ⃝ ⃝ ⃝

- - 1. Please identify an instance in any of your extracurricular activities where you recognized issues discussed in this course coming into play. If you do recall a scenario, please describe the ways in which you will solve these issues based on our course.

# Student Response Data

## Course Feedback Survey

| **Response** | **S1** | **S2** | **S3** | **S4** |
| --- | --- | --- | --- | --- |
| **Strongly disagree** | 0% (n = 0) | 0% (n = 0) | 0% (n = 0) | 0% (n = 0) |
| **Disagree** | 0% (n = 0) | 4.8% (n=1) | 0% (n = 0) | 0% (n = 0) |
| **Neutral** | 0% (n = 0) | 4.8% (n=1) | 0% (n = 0) | 0% (n = 0) |
| **Agree** | 29% (n=6) | 24% (n=5) | 14% (n=3) | 14% (n=3) |
| **Strongly agree** | 71% (n=15) | 67% (n=14) | 86% (n=18) | 86% (n=18) |

Table S1: Student responses to the course feedback survey (n = 21). Percentages represent the proportion of students who chose the particular response out of all students who responded to the statement.

## Pre- and Post-course Attitude Assessments

### Statement Response Pre-course Attitude Assessment

**n = 50**

### Post-course Attitude Assessment n = 8

| Strongly disagree | 0% (n=0) | 0% (n=0) |
| --- | --- | --- |
| Disagree | 14% (n=7) | 0% (n=0) |
| **S1** Neutral | 28% (n=14) | 13% (n=1) |
| Agree | 42% (n=21) | 38% (n=3) |
| Strongly Agree | 14% (n=7) | 50% (n=4) |
| (No Answer) | 2% (n = 1) | 0% (n = 0) |
| Strongly disagree | 0% (n=0) | 0% (n=0) |
| Disagree | 4% (n=2) | 0% (n=0) |
| **S2** Neutral | 10% (n=5) | 0% (n=0) |
| Agree | 54% (n=27) | 25% (n=2) |
| Strongly Agree | 30% (n=15) | 75% (n=6) |
| (No Answer) | 2% (n = 1) | 0% (n = 0) |
| Strongly disagree | 0% (n=0) | 0% (n=0) |
| Disagree | 30% (n=15) | 0% (n=0) |
| **S3** Neutral | 22% (n=11) | 38% (n=3) |
| Agree | 42% (n=21) | 38% (n=3) |
| Strongly Agree | 4% (n=2) | 25% (n=2) |
| (No Answer) | 2% (n = 1) | 0% (n = 0) |
| Strongly disagree | 4% (n=2) | 0% (n=0) |
| Disagree | 22% (n=11) | 0% (n=0) |
| **S4** Neutral | 28% (n=14) | 38% (n=3) |
| Agree | 40% (n=20) | 25% (n=2) |
| Strongly Agree | 4% (n=2) | 38% (n=3) |
| (No Answer) | 2% (n = 1) | 0% (n = 0) |

Table S2: Student responses on Pre-course and Post-course Attitude Assessments before taking the course and after taking the course. Empty responses were excluded from this study. 49 out of the 50 submissions on the Pre-course Attitude Assessment were analyzed.

# Qualitative Themes & Exemplar Data

| **Parent code** | **Child code** | **Exemplar quote** |
| --- | --- | --- |
| Awareness & introspection | Pursuing more education | *I plan to further my education in health inequity by*  *pursuing a masters of public health.* (Participant QCG) |
|  | Educating others | *I think for me, it is important to educate others (colleagues,*  *friends, family) to raise awareness.* (Participant DYN) |
|  | Providing gender-affirming care | *Learning more about gender affirming healthcare since I honestly haven’t heard of this term until I took this course*  (Participant 0CR) |
|  | Cultural competency | *Be more mindful in terms of cultural competence and*  *understanding my role in being better in it* (Participant 1GV) |
|  | Awareness impacting future practice | *Consider how I can minimize unequal outcomes in my*  *own provision of healthcare* (Participant 5LG) |
| Nature of barriers | Bias as a barrier | *To keep in mind that I am going to still make mistakes based*  *on biases I may have within myself.* (Participant 1GV) |
|  | Others disagree | *I would love to apply what I have learned, but if my colleagues are not in agreeance, then it might be difficult.*  (Participant DYN) |
|  | Legal & legislative | *Preexisting policies and current laws can present potential*  *barriers.* (Participant 7IH) |
|  | Organizational barriers | *So many barriers at the institutional level seem difficult to change as just one individual, and I bet it’s easy to fall in line with what is ”typical” and already readily practiced.*  (Participant YFK) |
|  | Financial/profit motive | *Healthcare system in general is more of a business rather than wanting to actually provide help to patients so the unequal access to healthcare will prevent me from applying*  *what I learned* (Participant N0B) |
| Likes & recommendations | Self-paced as plus | *The short videos were great to watch in bursts or*  *individually based on how much time you had to dedicate to the course that day.* (Participant Z9Q) |
|  | Case studies as plus | *I liked the case studies provided to reinforce the information*  *shared in the modules.* (Participant ZZX) |
|  | Adding discussion | *I think more guided discussions would be beneficial or virtual coffee talks? Something in place to ask more questions or engage with others taking the course.*  (Participant KXZ) |
|  | Informative as plus | *It was all very informative and helpful.* (Participant I32) |
|  | Amount of resources (+/-) | *One possible suggestion on how to improve the course for*  *future students is less recommended reading, but that’s my suggestion.* (Participant LXO) |
|  | Slide design (+/-) | *I would just suggest for the coordinators to work together to*  *have a more cohesive layout of the slides as some slides had no words while others had so many words.* (Participant N0B) |

Table S3: Qualitative themes and exemplar data from course feedback surveys.
